# New provincial records of the genus *Limnonectes* (Amphibia, Anura, Dicroglossidae) from South Central, Vietnam

**DOI:** 10.3897/BDJ.13.e146098

**Published:** 2025-04-30

**Authors:** Vien Hong Thi Nguyen, Tien Quang Phan, Dang Trong Do, Truong Quang Nguyen, Thomas Ziegler, Cuong The Pham, Anh Van Pham

**Affiliations:** 1 Faculty of Resources and Environment, University of Sciences, Thai Nguyen University, Tan Thinh Ward, Thai Nguyen City, Vietnam Faculty of Resources and Environment, University of Sciences, Thai Nguyen University, Tan Thinh Ward Thai Nguyen City Vietnam; 2 Faculty of Environmental Sciences, University of Science, Vietnam National University, Hanoi, 334 Nguyen Trai Road, Hanoi, Vietnam Faculty of Environmental Sciences, University of Science, Vietnam National University, Hanoi, 334 Nguyen Trai Road Hanoi Vietnam; 3 Institute of Biology, Vietnam Academy of Science and Technology, 18 Hoang Quoc Viet Road, Cau Giay District, Hanoi, Vietnam Institute of Biology, Vietnam Academy of Science and Technology, 18 Hoang Quoc Viet Road, Cau Giay District Hanoi Vietnam; 4 Phu Yen University, 01 Nguyen Van Huyen Road, Tuy Hoa City, Phu Yen Province, Vietnam Phu Yen University, 01 Nguyen Van Huyen Road, Tuy Hoa City Phu Yen Province Vietnam; 5 Graduate University of Science and Technology, Vietnam Academy of Science and Technology, 18 Hoang Quoc Viet Road, Cau Giay District, Hanoi, Vietnam Graduate University of Science and Technology, Vietnam Academy of Science and Technology, 18 Hoang Quoc Viet Road, Cau Giay District Hanoi Vietnam; 6 Cologne Zoo, Cologne, Germany Cologne Zoo Cologne Germany; 7 Institute of Zoology, University of Cologne, Cologne, Germany Institute of Zoology, University of Cologne Cologne Germany

**Keywords:** distribution, *Limnonectes*, new records, morphology, taxonomy

## Abstract

**Background:**

*Limnonectes* is one of the poorly-known genera of amphibians in Vietnam. In the herpetofaunal list of Vietnam in 2009, only five species of the genus *Limnonectes* were recorded from the country. Recently, four new species (*L.nguyenorum*, *L.quangninhensis*, *L.kiziriani* and *L.phuyenensis*) and three new country records (*L.gyldenstolpei*, *L.fastigatus* and *L.kohchangae*) were reported from Vietnam.

**New information:**

Based on recent field-work in South Central of Vietnam, we report four new provincial records of the genus *Limnonectes* from Phu Yen, Khanh Hoa, Binh Thuan and Gia Lai Provinces, namely *L.dabanus*, *L.fastigatus*, *L.limborgi* and *L.phuyenensis*. In addition, morphological data and ecological notes of the aforementioned species are provided.

## Introduction

The genus *Limnonectes* Fitzinger, 1843 currently contains 93 recognised species with a wide distribution in Asia, from eastern and southern China, eastwards to Japan, throughout Indochina and southwards to Malaysia, Indonesia, the Philippines and New Guinea (Frost 2025). More than a decade ago, Nguyen et al. (2009) recorded only five species of *Limnonectes* from Vietnam, i.e. *L.dabanus* (Smith, 1922), *L.limborgi* (Sclater, 1892) (as *L.hascheanus*, 1870), *L.bannaensis* Ye, Fei, Xie & Jiang, 2007 (as *L.kuhlii*, 1838), *L.khammonensis* (Smith, 1929) and *L.poilani* (Bourret, 1942). Since that time, a total of 11 species of the genus have been documented from this country (Frost 2025), including new records such as *L.gyldenstolpei* (Andersson, 1916) by Luu et al. (2013), *L.kohchangae* (Smith) by Nguyen et al. (2022) and *L.fastigatus* Stuart, Schoen, Nelson, Maher, Neang, Rowley & McLeod by Pham et al. (2022). Remarkably, four new species were described from Vietnam, viz. *L.kiziriani* Pham, Le, Ngo, Ziegler & Nguyen, *L.nguyenorum* McLeod, Kurlbaum & Hoang, *L.phuyenensis* Pham, Do, Le, Ngo, Nguyen, Ziegler & Nguyen and *L.quangninhensis* Pham, Le, Nguyen, Ziegler, Wu & Nguyen (McLeod et al. 2015, Pham et al. 2017, Pham et al. 2018, Pham et al. 2020).

*Limnonectesdabanus* (Smith) and *L.limborgi* (Sclater) are listed as Least Concern in the IUCN Red List (IUCN 2024), while *L.fastigatus* and *L.phuyenensis* were newly described in 2020 (Pham et al. 2020, Stuart et al. 2020). *Limnonectesfastigatus* is known only from Ratanakiri Province of Cambodia and Kon Tum Province of Vietnam, while *L.phuyenensis* is known only from Tay Hoa District, Phu Yen Province of Vietnam (Frost 2025). *Limnonectesdabanus* is known from very few records in south Vietnam (Frost 2025), while *L.limborgi* is a widely distributed species known from Thailand, Myanmar, Malaysia, Laos, Cambodia and Vietnam (from Lao Cai and Quang Ninh Provinces southwards to Dong Nai and Kien Giang Provinces) (Frost 2025).

Based on our recent fieldwork in Vietnam between 2022 and 2024, we herein report four new provincial records of the genus *Limnonectes* from Vietnam: *L.dabanus* from Binh Thuan Province, *L.fastigtus* from Gia Lai Province, *L.limborgi* from Khanh Hoa and Phu Yen Provinces and *L.phuyenensis* from Khanh Hoa Province.

## Materials and methods

**Sampling**

Field surveys were conducted in three localities in South Central Vietnam: in Takou Nature Reserve, Binh Thuan Province in March 2022; in Van Ninh District, Khanh Hoa Province in May 2023 and in October 2024; in Son Hoa District, Phu Yen Province in October 2024; and in Kon Cha Rang Nature Reserve, Gia Lai Province in May 2024.

Frogs were collected by hand between 19:00 and 22:00 h. After taking photographs, frogs were anaesthetised and euthanised in a closed vessel with a piece of cotton wool containing ethyl acetate (Simmons 2015), fixed in 80% ethanol for five hours and then transferred to 70% ethanol for permanent storage. Specimens referred to in this paper are deposited in the collection of the Institute of Biology, Hanoi, Vietnam.

**Morphological characters**

Measurements were taken with a digital caliper to the nearest 0.1 mm. Morphological terminology followed Pham et al. (2018). Abbreviations are as follows: SVL: snout-vent length; HL: head length from posterior corner of mandible to tip of snout; HW: maximum head width, at the angle of jaws; IND: internarial distance; RL: distance from anterior corner of eye to tip of snout; NS: distance from anterior edge of nostril to tip of snout; EN: distance from anterior corner of eye to posterior edge of nostril; ED: eye diameter; IOD: minimum distance between upper eyelids; UEW: maximum width of upper eyelid; TD: maximum tympanum diameter; UAL: upper arm length from axilla to elbow; FAL: fore-arm length from elbow to tip of third finger; FeL: thigh length, from vent to knee; TbL shank length; TbW: tibia width; FoL: foot length (from tarsus to tip of fourth toe). Sex was determined by the presence of nuptial pads in males and based on gonadal inspection.

**Molecular analysis**

The tissue samples of *Limnonectes* were extracted using QIAamp DNA Mini Kit (Qiagen, Germany) following protocols by the manufacturer. Total DNA was then amplified by HotStar Taq Mastermix (Qiagen, Germany). The standard PCR conditions were 95^o^C for 15 min to active Taq; 35 cycles at 95^o^C for 30s, 50^o^C for 45s, 72^o^C for 1 min; and a final elongation at 72^o^C for 10 min. The PCR volume contained 2 µl of each primer at 10 µmol/µl, 5 µl water, 10 µl of Mastermix and 2 µl DNA template. The primers used to amplify a fragment of the mitochondrial DNA 16S gene were AR (5’-CGCCTGTTTATCAAAAACAT-3’) and BR (5’-CCGGTCTGAACTCAGATCACGT-3’) (Palumbi et al. 1991). PCR products were visualised using electrophoresis through a 1% agarose gel, marker 1kb, in 1X TBE and stained with ethidium bromide and photographed under UV light. Successful amplifications were purified using GeneJet PCR Purification Kit (ThermoFisher Scientific, Lithuania). Cleaned PCR products were sent to 1st Base (Malaysia) for sequencing in both directions using the same primers. Sequences were validated with Sequencher v.4.10 (Gene Codes, Ann Arbor, MI) with default setting and compared with data available on GenBank using BLAST Tool as implemented in the National Center for Biotechnology Information (NCBI, https://blast.ncbi.nlm.nih.gov/).

## Taxon treatments

### Limnonectes dabanus

(Smith, 1922)

DC9C04D9-2972-506C-9E79-103BEE376C69

#### Materials

- **Type status:** Other material. **Occurrence:** catalogNumber: IEBR A.6337 (field No. BT.2022.18); individualCount: 1; sex: male; lifeStage: adult; occurrenceID: 69E304D0-543C-547E-AAD1-0EF3841FAFA5; **Taxon:** scientificNameID: *Limnonectesdabanus*; scientificName: *Limnonectesdabanus*; class: Amphibia; order: Anura; family: Dicroglossidae; genus: Limnonectes; specificEpithet: *dabanus*; scientificNameAuthorship: Smith, 1922; **Location:** country: Vietnam; countryCode: VN; stateProvince: Binh Thuan; county: Binh Thuan; municipality: Ham Thuan Nam; locality: Ta Kou Nature Reserve; verbatimElevation: 350; verbatimLatitude: 10°48'43.6"N; verbatimLongitude: 107°53'38.7"E; verbatimCoordinateSystem: WGS84; **Event:** eventDate: March 25, 2022; eventRemarks: collected by AV Pham; **Record Level:** language: en; collectionCode: Amphibians; basisOfRecord: PreservedSpecimen
- **Type status:** Other material. **Occurrence:** catalogNumber: IEBR A.6338 (field No. BT.2022.19); individualCount: 1; sex: male; lifeStage: adult; occurrenceID: 20BD3F99-C308-525D-9D9E-D932E31E82C2; **Taxon:** scientificNameID: *Limnonectesdabanus*; scientificName: *Limnonectesdabanus*; class: Amphibia; order: Anura; family: Dicroglossidae; genus: Limnonectes; specificEpithet: dabanus; scientificNameAuthorship: Smith, 1922; **Location:** country: Vietnam; countryCode: VN; stateProvince: Binh Thuan; county: Binh Thuan; municipality: Ham Thuan Nam; locality: Ta Kou Nature Reserve; verbatimElevation: 350; verbatimLatitude: 10°48'43.6"N; verbatimLongitude: 107°53'38.7"E; verbatimCoordinateSystem: WGS84; **Event:** eventDate: March 25, 2022; eventRemarks: collected by AV Pham; **Record Level:** language: en; collectionCode: Amphibians; basisOfRecord: PreservedSpecimen
- **Type status:** Other material. **Occurrence:** catalogNumber: IEBR A.6339 (field No. BT.2022.20); individualCount: 1; sex: female; lifeStage: adult; occurrenceID: CAFB0FB5-8FC1-5DFE-8B30-7701C554C1BE; **Taxon:** scientificNameID: *Limnonectesdabanus*; scientificName: *Limnonectesdabanus*; class: Amphibia; order: Anura; family: Dicroglossidae; genus: Limnonectes; specificEpithet: *dabanus*; scientificNameAuthorship: Smith, 1922; **Location:** country: Vietnam; countryCode: VN; stateProvince: Binh Thuan; county: Binh Thuan; municipality: Ham Thuan Nam; locality: Ta Kou Nature Reserve; verbatimElevation: 350; verbatimLatitude: 10°48'43.6"N; verbatimLongitude: 107°53'38.7"E; verbatimCoordinateSystem: WGS84; **Event:** eventDate: March 25, 2022; eventRemarks: collected by AV Pham; **Record Level:** language: en; collectionCode: Amphibians; basisOfRecord: PreservedSpecimen
- **Type status:** Other material. **Occurrence:** catalogNumber: IEBR A.6340 (field No. BT.2022.21); individualCount: 1; sex: female; lifeStage: adult; occurrenceID: F74F9FE9-277E-570C-BFD3-8B4180AE086F; **Taxon:** scientificNameID: *Limnonectesdabanus*; scientificName: *Limnonectesdabanus*; class: Amphibia; order: Anura; family: Dicroglossidae; genus: Limnonectes; specificEpithet: *dabanus*; scientificNameAuthorship: Smith, 1922; **Location:** country: Vietnam; countryCode: VN; stateProvince: Binh Thuan; county: Binh Thuan; municipality: Ham Thuan Nam; locality: Ta Kou Nature Reserve; verbatimElevation: 350; verbatimLatitude: 10°48'43.6"N; verbatimLongitude: 107°53'38.7"E; verbatimCoordinateSystem: WGS84; **Event:** eventDate: March 25, 2022; eventRemarks: collected by AV Pham; **Record Level:** language: en; collectionCode: Amphibians; basisOfRecord: PreservedSpecimen

#### Description

One sequence of 560 bps (16S gene) from specimen IEBR A.6338 (GenBank accession number PV444292) of *Limnonectes* specimen from Ta Kou Nature Reserve, Binh Thuan Province was similar (99.5%) to the available sequence of *L.dabanus* (accession number MK688610) on GenBank.

**Figure 1.**
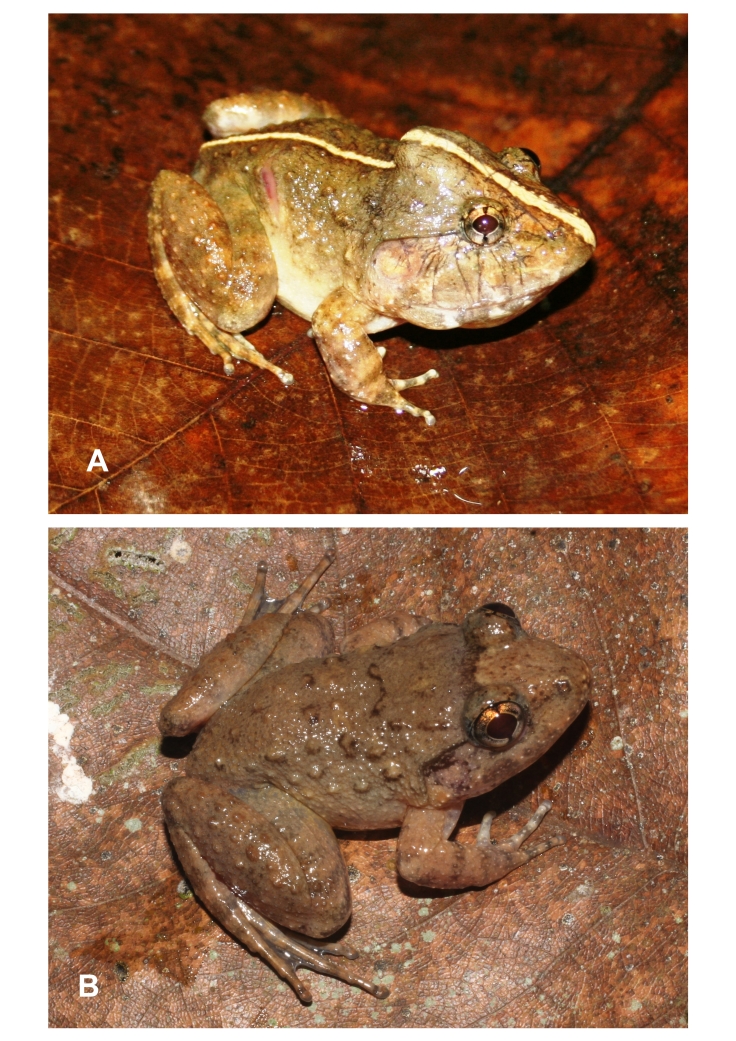
*Limnonectesdabanus* from Ta Kou Nature Reserve, Binh Thuan Province: **A** male, IEBR A.6337; **B** female, IEBR A.6337 .

Morphological characters of specimens from Binh Thuan Province agreed with the descriptions of Bourret (1942), Lambertz et al. (2014) and Pham et al. (2019). Size medium, males slightly larger than females (SVL 52.4-53.3 mm in males, n = 2; 44.7-44.9 mm in females, n = 2); head width broader than long (HL 23.5-27.3 mm, HW 23.8-27.6 mm in males; 18.2-18.3 mm, 18.3-18.5 mm in females); snout round anteriorly in dorsal view; rostral length greater than eye diameter (RL 8.5-9.5 mm, ED 5.7-5.9 mm in males; 6.8-7.1 mm, 5.1-5.2 mm in females); tympanum distinct, smaller than the eye diameter (TD/ED 0.90-0.91 in males, 0.85-0.88 in females); vomerine teeth in two oblique ridges; tongue cordiform, deeply notched posteriorly; lower jaw with two tooth, well developed; lacking vocal sacs in males (Table 1and Fig. 1).

**Figure 2.**
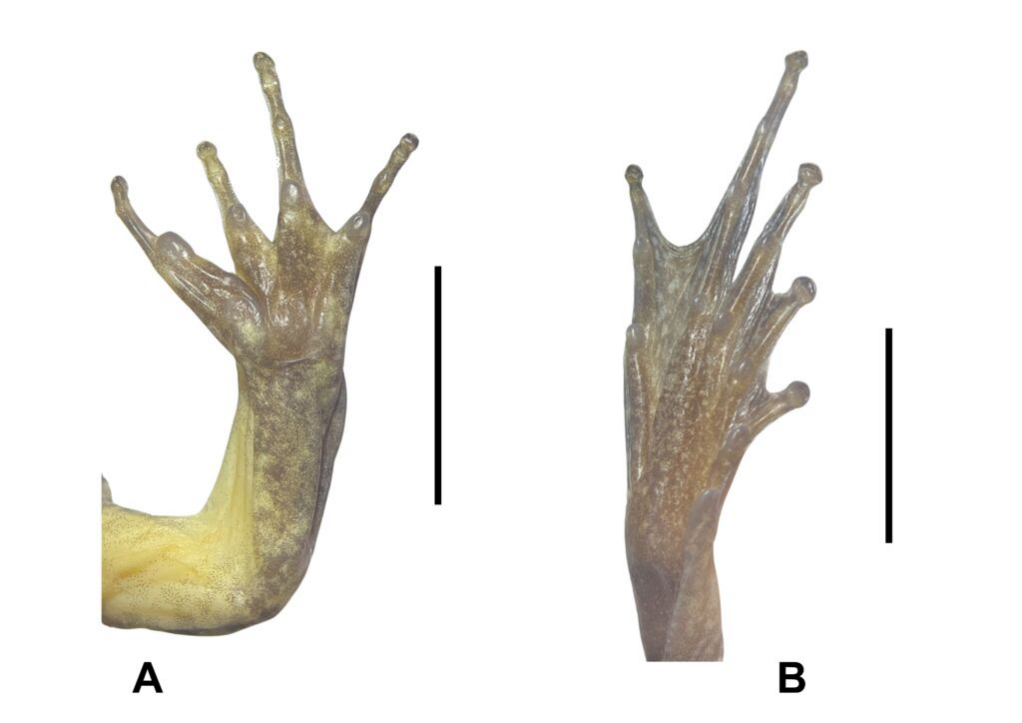
**A** lower right hand of *Limnonectesdabanus* (male, IEBR A.6337); **B** lower right foot of *Limnonectesdabanus* (male, IEBR A.6337).

Arms short; fingers free of webbing; tips of fingers blunt, not expanded; finger I with nuptial pad, without minute spines in males. Tibia length shorter than thigh; tips of toes blunt, round; webbing well developed, formula I0–1/3II0–1/4III0–1IV1–0V; tibio-tarsal articulation reaching to the eye (Fig. 2).

Skin texture in life: Dorsal surface of head smooth with a swollen flap in males; dorsal surface of body and flanks with tubercles; supratympanic fold distinct; dorsal surface of limbs and thighs smooth; dorsal surface of tibia with small tubercles; throat, chest, belly and ventral surface of thighs smooth.

Colouration in life: Dorsal surface of head, body and flanks light brown with dark brown marking; dorsal surface of fore- and hind-limbs light brown with dark crossbars; ventral surface of limbs, throat, chest and belly white (Fig. 1).

#### Distribution

In Vietnam, this species has been reported from Dak Lak, Dak Nong, Lam Dong, Dong Nai, Binh Dinh, Phu Yen, Ninh Thuan and Khanh Hoa Provinces (Do et al. 2017, Pham et al. 2019, Frost 2025). Elsewhere, the species is known from Cambodia (Frost 2025). This is the first record of *L.dabanus* from Binh Thuan Province.

#### Ecology

The frogs were found between 21:00 and 22:30 h in rocky streams. Surrounding habitat consisted of mixed secondary forest composed of small to medium hardwoods and shrubs. Air temperature was 25–30°C and relative humidity was 55–70%.

### Limnonectes fastigatus

Stuart, Schoen, Nelson, Maher, Neang, Rowley & McLeod, 2020

6C9FE4F4-E132-594E-B280-F087C030989A

#### Materials

- **Type status:** Other material. **Occurrence:** catalogNumber: IEBR A.6341 (field No. KCR 2024.28); individualCount: 1; sex: male; lifeStage: adult; occurrenceID: 9ED2E779-A2ED-5DAF-87B6-CA1514E9868E; **Taxon:** scientificNameID: *Limnonectesfastigatus*; scientificName: *Limnonectesfastigatus*; class: Amphibia; order: Anura; family: Dicroglossidae; genus: Limnonectes; specificEpithet: *fastigatus*; scientificNameAuthorship: Stuart, Schoen, Nelson, Maher, Neang, Rowley, and McLeod, 2020; **Location:** country: Vietnam; countryCode: VN; stateProvince: Gia Lai; county: Gia Lai; locality: Kon Cha Rang Nature Reserve; verbatimElevation: 937 m; verbatimLatitude: 14°54.042'N; verbatimLongitude: 108°55.376'E; **Event:** eventDate: April 25, 2024; eventRemarks: collected by TQ Phan and HTL Le; **Record Level:** language: en; collectionCode: Amphibians; basisOfRecord: PreservedSpecimen
- **Type status:** Other material. **Occurrence:** catalogNumber: IEBR A.6342 (field No. KCR 2024.53); individualCount: 1; sex: male; lifeStage: adult; occurrenceID: 694FD8A2-EECD-5164-A083-9F03C8F31082; **Taxon:** scientificNameID: *Limnonectesfastigatus*; scientificName: *Limnonectesfastigatus*; class: Amphibia; order: Anura; family: Dicroglossidae; genus: Limnonectes; specificEpithet: *fastigatus*; scientificNameAuthorship: Stuart, Schoen, Nelson, Maher, Neang, Rowley, and McLeod, 2020; **Location:** country: Vietnam; countryCode: VN; stateProvince: Gia Lai; county: Gia Lai; locality: Kon Cha Rang Nature Reserve; verbatimElevation: 937 m; verbatimLatitude: 14°54.042'N; verbatimLongitude: 108°55.376'E; **Event:** eventDate: April 25, 2024; eventRemarks: collected by TQ Phan and HTL Le; **Record Level:** language: en; collectionCode: Amphibians; basisOfRecord: PreservedSpecimen

#### Description

One sequence of 560 bps (16S gene) from specimen IEBR A.6341 (GenBank accession number PV444293) of *Limnonectes* specimen from Kon Cha Rang Nature Reserve, Gia Lai Province was similar (98.2%) to the available sequence of *L.fastigatus* (accession number MT459155) on GenBank.

**Figure 3.**
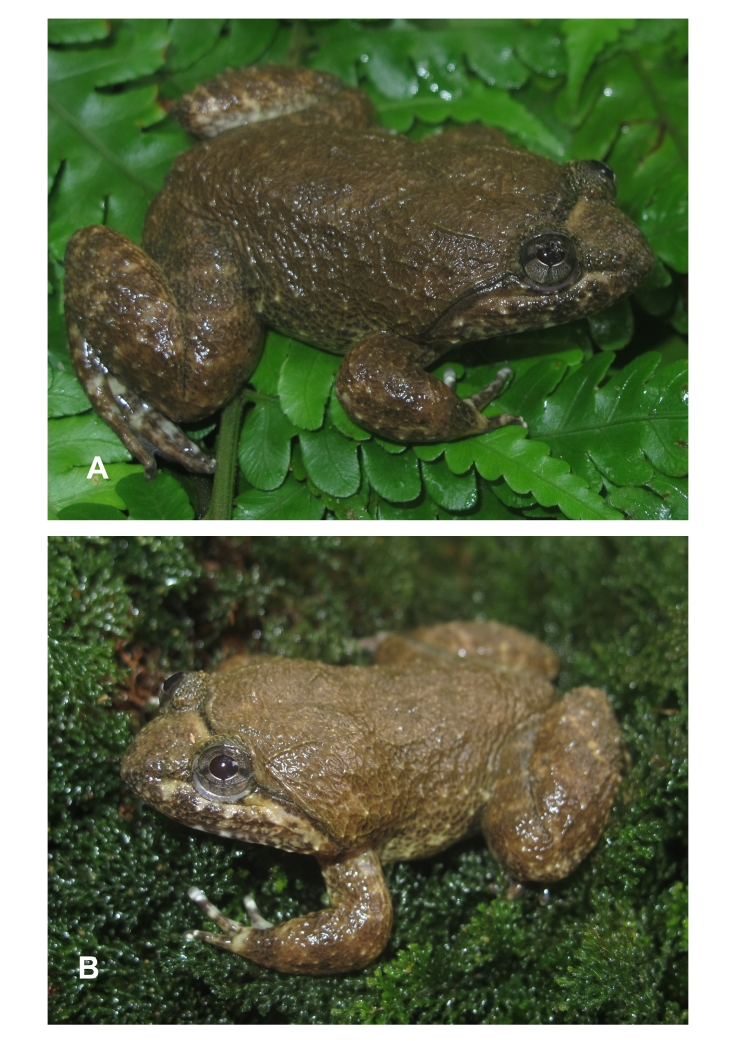
*Limnonectesfasstigatus* from Kon Cha Rang Nature Reserve, Gia Lai Province: **A** male, IEBR A.6341; **B** male, IEBR A.6342.

Morphological characters of specimens from Gia Lai Province agreed with the descriptions of Stuart et al. (2020) and Pham et al. (2022). Size large (SVL 58.9-68.6 mm in males, n = 2), habitus robust with moderately enlarged head (HL/SVL 0.43-0.45, HW/SVL 0.46-0.48 in males); head width broader than long (HL 26.3-29.8 mm, HW 27.1-32.6 mm, n = 2); snout round anteriorly in dorsal view, projecting beyond lower jaw; nostril lateral, about mid-way between snout tip and eye (NS 4.1-4.8 mm, EN 4.4-5.4 mm in males); canthus rostralis indistinct; loreal region oblique and slightly concave; rostral length greater than eye diameter (RL 8.5-10.2 mm, ED 7.1-8.0 mm in males); tympanum invisible; vomerine teeth in two oblique ridges; tongue cordiform, notched posteriorly; lower jaw with two tooth-like, odontoid processes robust thin and elongate with rounded tips; lacking vocal sacs in males (Table 1 and Fig. 3).

**Figure 4.**
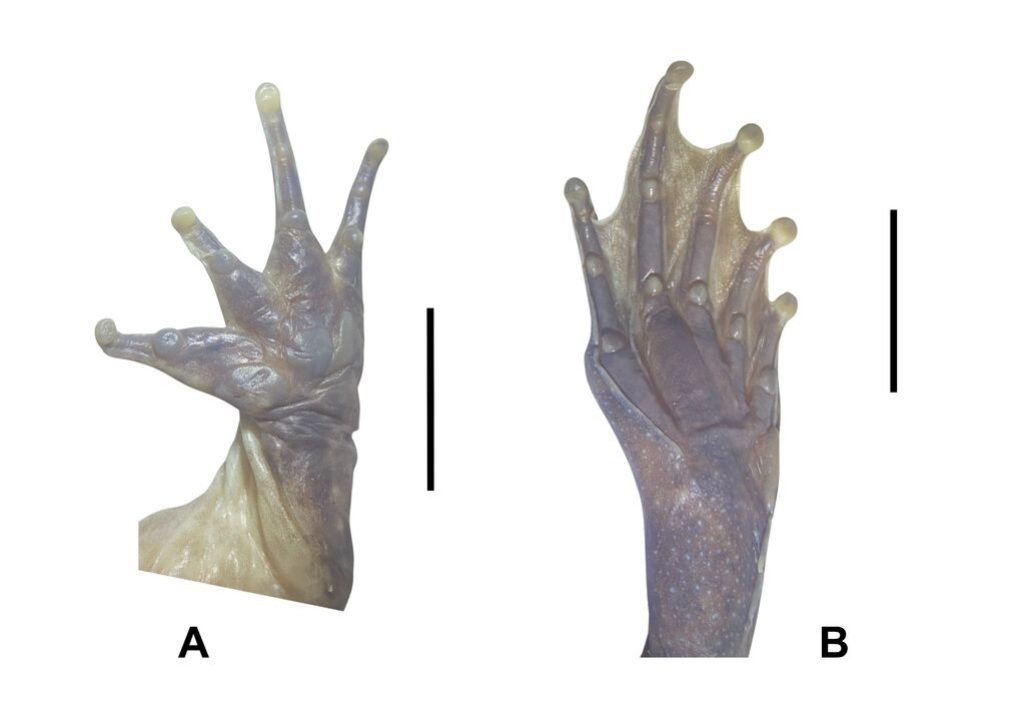
**A** lower right hand of *Limnonectesfastigatus* (male, IEBR A.6341); **B** lower right foot of *Limnonectesfastigatus* (male, IEBR A.6341).

Arms short; upper arm length (UAL 11.8-14.3 mm in males), forearm length (FAL 23.1-29.1 mm in males); fingers free of webbing; dermal ridge on sides of fingers II and III; tips of fingers blunt, not expanded; inner metatarsal tubercle large, oval; outer metatarsal tubercle small, elongate; finger I of males with nuptial pad, composed of minute spines on the dorsal surface and medial edge (Fig. 4).

Tibia length shorter than thigh length (FeL 27.2-31.1 mm, TbL 26.1-29.8 mm in males), longer than wide (TbL/TbW 2.33-2.42 in males); tips of toes blunt, slightly round; toes webbed to the middle of terminal phalanx at base of toe pad; webbing well developed; dermal ridge on outer sides of toes I and V; inner metatarsal tubercle elongate; outer metatarsal tubercle absent; tibio-tarsal articulation reaching behind the eye (Fig. 4).

Skin texture in life. Dorsal surface of head and body crenulate; small tubercles on the upper eyelid; flanks, around cloaca and dorsal surface of limbs and thighs with small tubercles; supratympanic fold distinct, extending from eye to angle of the jaw; dorsolateral fold absent; dorsal surface of tibia and foot distinctly tuberculate, covered with moderately dense, small, tubercles; throat, chest, belly and ventral surface of thighs smooth.

Colouration in life. Iris light brass with dark grey-brown lines bisecting eye vertically and horizontally through pupil; head with a pale yellow bar in the anterior interorbital region and a narrow dark brown bar in the posterior interorbital region; dorsum and flanks with dark grey-brown marking; lips with dark bars; supratympanic fold black; dorsal surface of limbs irregular, yellowish-brown with dark crossbars; ventral surface of limbs, throat and chest white with brown markings; belly immaculate white; toe webbing brown; the tip of fingers and toes white (Fig. 3).

#### Distribution

In Vietnam, this species was recorded in Kon Tum Province (Pham et al. 2022). Elsewhere, the species is known from Cambodia (Stuart et al. 2020). This is the first record of *L.fastigatus* from Gia Lai Province.

#### Ecology

The frogs were found between 19:00 and 23:00 h in the water of rocky streams or on the ground. The surrounding habitat was a mixed secondary evergreen forest of the large, medium and small hardwoods and shrubs. Air temperature was 18–28°C and relative humidity was 76–86%.

### Limnonectes limborgi

(Sclater, 1892)

7923D734-3D46-57BB-AAC2-185DAEEBD435

#### Materials

- **Type status:** Other material. **Occurrence:** catalogNumber: IEBR A.6343 (field No. PY.2024.1); individualCount: 1; sex: male; lifeStage: adult; occurrenceID: C8FDFB4E-4537-5572-99CD-53CDBFCAFBE0; **Taxon:** scientificNameID: *Limnonecteslimborgi*; scientificName: *Limnonecteslimborgi*; class: Amphibia; order: Anura; family: Dicroglossidae; genus: Limnonectes; specificEpithet: *limborgi*; scientificNameAuthorship: Sclater, 1892; **Location:** country: Vietnam; countryCode: VN; stateProvince: Phu Yen; county: Son Hoa District; municipality: Son Long Commune; verbatimElevation: 368 m; verbatimLatitude: 13°20.339'N; verbatimLongitude: 109°08.981'E; **Event:** eventDate: October 12, 2024; eventRemarks: collected by CT Pham and DT Do; **Record Level:** language: en; collectionCode: Amphibians; basisOfRecord: PreservedSpecimen
- **Type status:** Other material. **Occurrence:** catalogNumber: IEBR A.6344 (field No. PY.2024.2); individualCount: 1; sex: female; lifeStage: adult; occurrenceID: 714A3A58-433F-55BC-B2BB-55EBEE717F1C; **Taxon:** scientificNameID: *Limnonecteslimborgi*; scientificName: *Limnonecteslimborgi*; class: Amphibia; order: Anura; family: Dicroglossidae; genus: Limnonectes; specificEpithet: *limborgi*; scientificNameAuthorship: Sclater, 1892; **Location:** country: Vietnam; countryCode: VN; stateProvince: Phu Yen; county: Son Hoa District; municipality: Son Long Commune; verbatimElevation: 368 m; verbatimLatitude: 13°20.339'N; verbatimLongitude: 109°08.981'E; **Event:** eventDate: October 12, 2024; eventRemarks: collected by CT Pham and DT Do; **Record Level:** language: en; collectionCode: Amphibians; basisOfRecord: PreservedSpecimen
- **Type status:** Other material. **Occurrence:** catalogNumber: IEBR A.6345 (field No. KH.2024.1); individualCount: 1; sex: female; lifeStage: adult; occurrenceID: 819AA51D-E863-54B5-87B4-A3BFBAF10E51; **Taxon:** scientificNameID: *Limnonecteslimborgi*; scientificName: *Limnonecteslimborgi*; class: Amphibia; order: Anura; family: Dicroglossidae; genus: Limnonectes; specificEpithet: *limborgi*; scientificNameAuthorship: Sclater, 1892; **Location:** country: Vietnam; countryCode: VN; stateProvince: Khanh Hoa; county: Van Ninh District; municipality: Van Da Commune; verbatimElevation: 440 m; verbatimLatitude: 12°78.35'N; verbatimLongitude: 109°17.534'E; **Event:** eventDate: October 12, 2024; eventRemarks: collected by CT Pham and DT Do; **Record Level:** language: en; collectionCode: Amphibians; basisOfRecord: PreservedSpecimen
- **Type status:** Other material. **Occurrence:** catalogNumber: IEBR A.6346 (field No. KH.2024.2); individualCount: 1; sex: female; lifeStage: adult; occurrenceID: F8133883-897B-5A88-87F4-ACD221760EFA; **Taxon:** scientificNameID: *Limnonecteslimborgi*; scientificName: *Limnonecteslimborgi*; class: Amphibia; order: Anura; family: Dicroglossidae; genus: Limnonectes; specificEpithet: *limborgi*; scientificNameAuthorship: Sclater, 1892; **Location:** country: Vietnam; countryCode: VN; stateProvince: Khanh Hoa; county: Van Ninh District; municipality: Van Da Commune; verbatimElevation: 440 m; verbatimLatitude: 12°78.35'N; verbatimLongitude: 109°17.534'E; **Event:** eventDate: October 12, 2024; eventRemarks: collected by CT Pham and DT Do; **Record Level:** language: en; collectionCode: Amphibians; basisOfRecord: PreservedSpecimen

#### Description

One sequence of 560 bps (16S gene) from specimen IEBR A.6343 (GenBank accession number PV444294) of *Limnonectes* specimen from Van Ninh District, Khanh Hoa Province was similar (100%) to the available sequence of *L.limborgi* (accession number GU934345) on GenBank.

**Figure 5.**
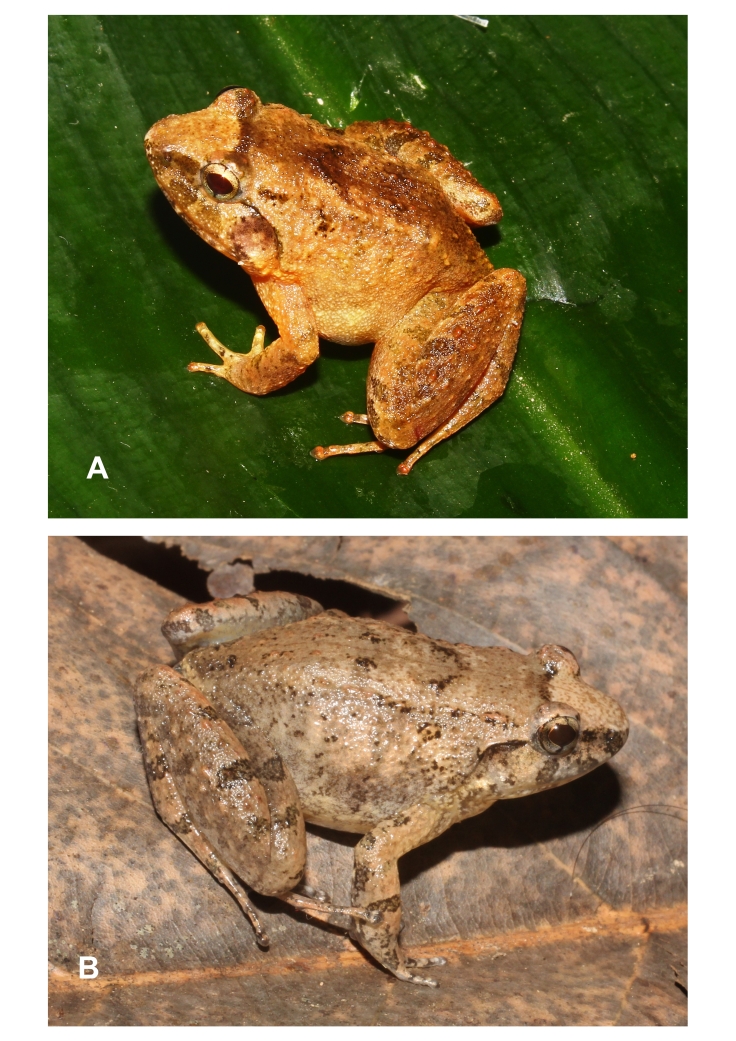
*Limnonecteslimborgi* from Phu Yen Province: **A** male, IEBR A.6343; **B** female, IEBR A.6344.

Morphological characters of specimens from Phu Yen and Khanh Hoa Provinces agreed with the descriptions of Bourret (1942), Inger and Stuart (2010) and Pham et al. (2019). Size small (SVL 36.4 mm in male, n = 1; 33.0-34.5 mm in females, n = 3), head slightly longer than wide (HL 15.5 mm, HW 15.0 mm in male; 12.5-14.2 mm, 12.3-13.7 mm in females); snout slightly pointed anteriorly in dorsal view; rostral length greater than eye diameter (RL 6.0 mm, ED 4.0 mm in male; 5.0-5.3 mm, 4.3-4.5 mm in females); tympanum visible, round, approximately 50% of eye diameter (TD 2.1 in male; 2.7-2.8 in females); vomerine teeth in two oblique ridges; tongue cordiforn, deeply notched posteriorly; vocal sacs absent in the male (Table 1 and Fig. 5).

**Figure 6.**
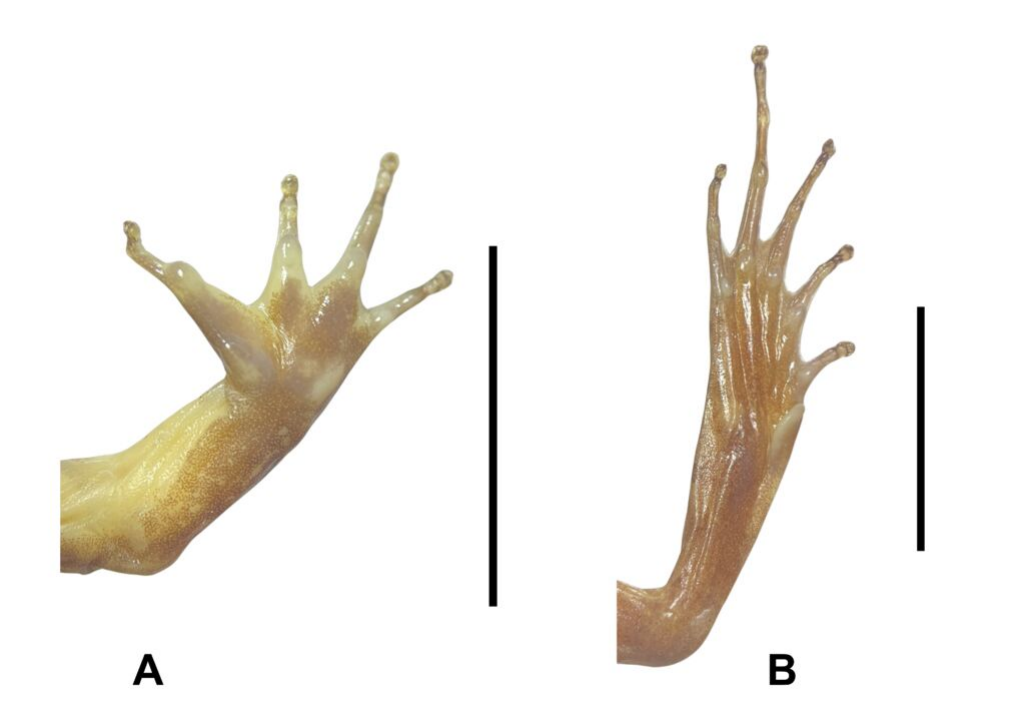
**A** lower right hand of *Limnonecteslimborgi* (male, IEBR A.6343); **B** lower right foot of *Limnonecteslimborgi* (male, IEBR A.6343).

Arms short and thin; fingers free of webbing; tips of fingers blunt, not expanded into discs; nuptial pad absent on finger I in males. Tibia and thigh short; tips of toes blunt, not expanded into discs; webbing formula I1–2II11/2–21/2III2–3IV31/2–11/2V; tibio-tarsal articulation reaching between eye and tip of snout (Fig. 6).

Skin texture in life: Dorsal surface of head and body with small tubercles; supratympanic fold distinct; dorsolateral folds present; ventral surface smooth.

Colouration in life: Dorsal surface light brown or light yellow with small dark spots; a dark cross bar between the eyes and a ᴧ-shaped mark between the shoulders; thighs and tibia with dark brown cross bars; ventral surface cream; throat cream with dark pattern (Fig. 5).

#### Distribution

In Vietnam, this species is known from Lao Cai in the north southwards to Dong Nai and Kien Giang Provinces (Pham et al. 2019, Le et al. 2021, Frost 2025). Elsewhere, the species has been reported from Lao PDR, Cambodia, Thailand and Myanmar (Frost 2025). This is the first record of *L.limborgi* from Phu Yen and Khanh Hoa Provinces.

#### Ecology

The frogs were found between 19:00 and 22:30 h on forest paths. Surrounding habitat consisted of mixed secondary forest composed of small to medium hardwoods and shrubs. Air temperature was 23–30°C and relative humidity was 65–81%.

### Limnonectes phuyenensis

Pham, Do, Le, Ngo, Nguyen, Ziegler & Nguyen, 2020

918A3956-1FF9-5BF5-BC05-A9206C707A96

#### Materials

- **Type status:** Other material. **Occurrence:** catalogNumber: IEBR A.6348 (field No. KH.2023.63); individualCount: 1; sex: male; lifeStage: adult; occurrenceID: 95430501-23BA-5A50-9D6C-7D02BB1C1A23; **Taxon:** scientificNameID: *Limnonectesphuyenensis*; scientificName: *Limnonectesphuyenensis*; class: Amphibia; order: Anura; family: Dicroglossidae; genus: Limnonectes; specificEpithet: *phuyenensis*; scientificNameAuthorship: Pham, Do, Le, Ngo, Nguyen, Ziegler & Nguyen, 2020; **Location:** country: Vietnam; countryCode: VN; stateProvince: Khanh Hoa; county: Van Ninh District; municipality: Van Da Commune; verbatimElevation: 319 m; verbatimLatitude: 12°46.317'N; verbatimLongitude: 109°10.473'E; **Event:** eventDate: May 26, 2023; eventRemarks: collected by CV Hoang and QT Phan; **Record Level:** language: en; collectionCode: Amphibians; basisOfRecord: PreservedSpecimen
- **Type status:** Other material. **Occurrence:** catalogNumber: IEBR A.6349 (field No. KH.2023.65); individualCount: 1; sex: male; lifeStage: adult; occurrenceID: 655DE0C9-6A02-52C2-A38E-944920FFE785; **Taxon:** scientificNameID: *Limnonectesphuyenensis*; scientificName: *Limnonectesphuyenensis*; class: Amphibia; order: Anura; family: Dicroglossidae; genus: Limnonectes; specificEpithet: *phuyenensis*; scientificNameAuthorship: Pham, Do, Le, Ngo, Nguyen, Ziegler & Nguyen, 2020; **Location:** country: Vietnam; countryCode: VN; stateProvince: Khanh Hoa; county: Van Ninh District; municipality: Van Da Commune; verbatimElevation: 319 m; verbatimLatitude: 12°46.317'N; verbatimLongitude: 109°10.473'E; **Event:** eventDate: May 26, 2023; eventRemarks: collected by CV Hoang and QT Phan; **Record Level:** language: en; collectionCode: Amphibians; basisOfRecord: PreservedSpecimen
- **Type status:** Other material. **Occurrence:** catalogNumber: IEBR A.6350 (field No. KH.2023.66); individualCount: 1; sex: male; lifeStage: adult; occurrenceID: 6F5DF39F-2A24-5814-9981-859BD1C2CCBE; **Taxon:** scientificNameID: *Limnonectesphuyenensis*; scientificName: *Limnonectesphuyenensis*; class: Amphibia; order: Anura; family: Dicroglossidae; genus: Limnonectes; specificEpithet: *phuyenensis*; scientificNameAuthorship: Pham, Do, Le, Ngo, Nguyen, Ziegler & Nguyen, 2020; **Location:** country: Vietnam; countryCode: VN; stateProvince: Khanh Hoa; county: Van Ninh District; municipality: Van Da Commune; verbatimElevation: 319 m; verbatimLatitude: 12°46.317'N; verbatimLongitude: 109°10.473'E; **Event:** eventDate: May 26, 2023; eventRemarks: collected by CV Hoang and QT Phan; **Record Level:** language: en; collectionCode: Amphibians; basisOfRecord: PreservedSpecimen
- **Type status:** Other material. **Occurrence:** catalogNumber: IEBR A.6351 (field No. KH.2023.67); individualCount: 1; sex: male; lifeStage: adult; occurrenceID: AE09C6AA-F2B6-5853-BDDA-DCE9A6C97410; **Taxon:** scientificNameID: *Limnonectesphuyenensis*; scientificName: *Limnonectesphuyenensis*; class: Amphibia; order: Anura; family: Dicroglossidae; genus: Limnonectes; specificEpithet: *phuyenensis*; scientificNameAuthorship: Pham, Do, Le, Ngo, Nguyen, Ziegler & Nguyen, 2020; **Location:** country: Vietnam; countryCode: VN; stateProvince: Khanh Hoa; county: Van Ninh District; municipality: Van Da Commune; verbatimElevation: 319 m; verbatimLatitude: 12°46.317'N; verbatimLongitude: 109°10.473'E; **Event:** eventDate: May 26, 2023; eventRemarks: collected by CV Hoang and QT Phan; **Record Level:** language: en; collectionCode: Amphibians; basisOfRecord: PreservedSpecimen
- **Type status:** Other material. **Occurrence:** catalogNumber: IEBR A.6352 (field No. KH.2023.64); individualCount: 1; sex: female; lifeStage: adult; occurrenceID: A9840ED2-569B-5EB1-BB9E-B05BE4E594F5; **Taxon:** scientificNameID: *Limnonectesphuyenensis*; scientificName: *Limnonectesphuyenensis*; class: Amphibia; order: Anura; family: Dicroglossidae; genus: Limnonectes; specificEpithet: *phuyenensis*; scientificNameAuthorship: Pham, Do, Le, Ngo, Nguyen, Ziegler & Nguyen, 2020; **Location:** country: Vietnam; countryCode: VN; stateProvince: Khanh Hoa; county: Van Ninh District; municipality: Van Da Commune; verbatimElevation: 371 m; verbatimLatitude: 12°46.314'N; verbatimLongitude: 109°10.441'E; **Event:** eventDate: May 26, 2023; eventRemarks: collected by CV Hoang and QT Phan; **Record Level:** language: en; collectionCode: Amphibians; basisOfRecord: PreservedSpecimen

#### Description

One sequence of 560 bps (16S gene) from specimen IEBR A.6348 (GenBank accession number PV444294) of *Limnonectes* specimen from Son Hoa District, Phu Yen Province was similar (99.8%) to the available sequence of *L.phuyenensis* (accession number MW222163) on GenBank.

**Figure 7.**
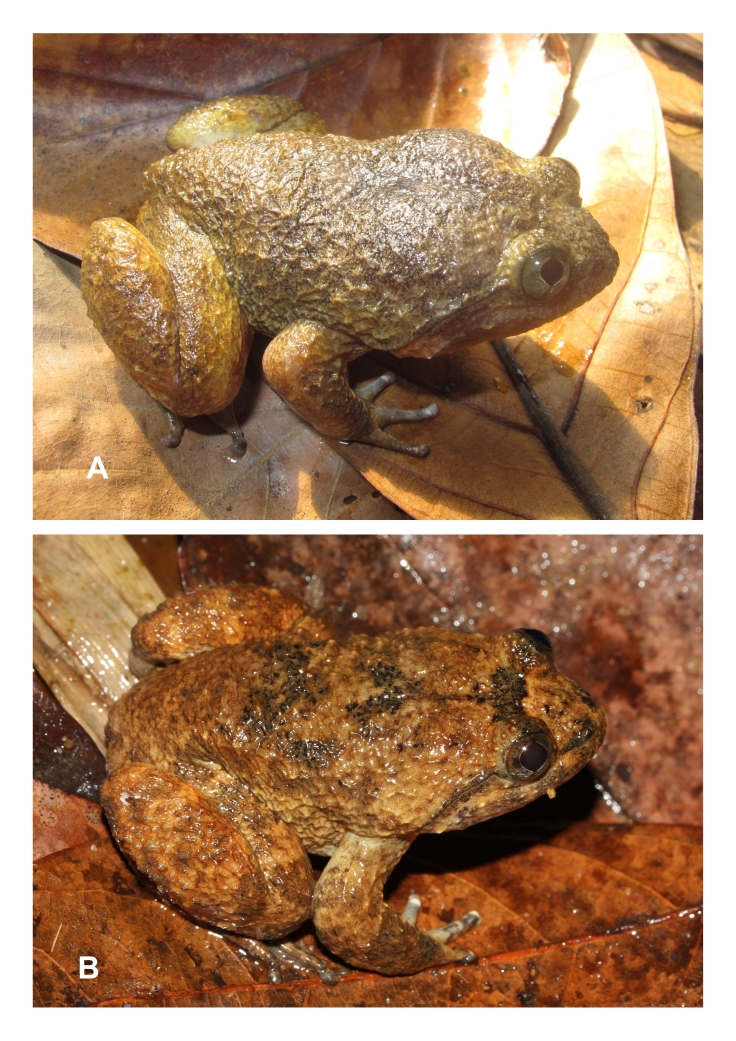
*Limnonectesphuyenensis* from Khanh Hoa Province: **A** male, IEBR A.6348; **B** female, IEBR A.6352.

Morphological characters of specimens from Khanh Hoa Province agreed with the description of Pham et al. (2020). Size medium (SVL 61.5-69.9 mm in males, n = 4; 45.8 mm in female, n = 1); head slightly broader than long (HL 28.8-32.2 mm, HW 29.7-33.3 mm, HL/HW 0.96 in males; 19.9 mm, 29.5 mm, HL/HW 0.97 in female); rostral length short (RL/SVL 0.14-0.15 in males, 0.15 in female), greater than eye diameter (RL 8.6-9.9 mm, ED 7.2-8.4 mm in males; 7.0 mm, 5.9 mm in females); tympanum invisible, vomerine teeth present; lacking vocal sacs in males (Table 1 and Fig. 7).

**Figure 8.**
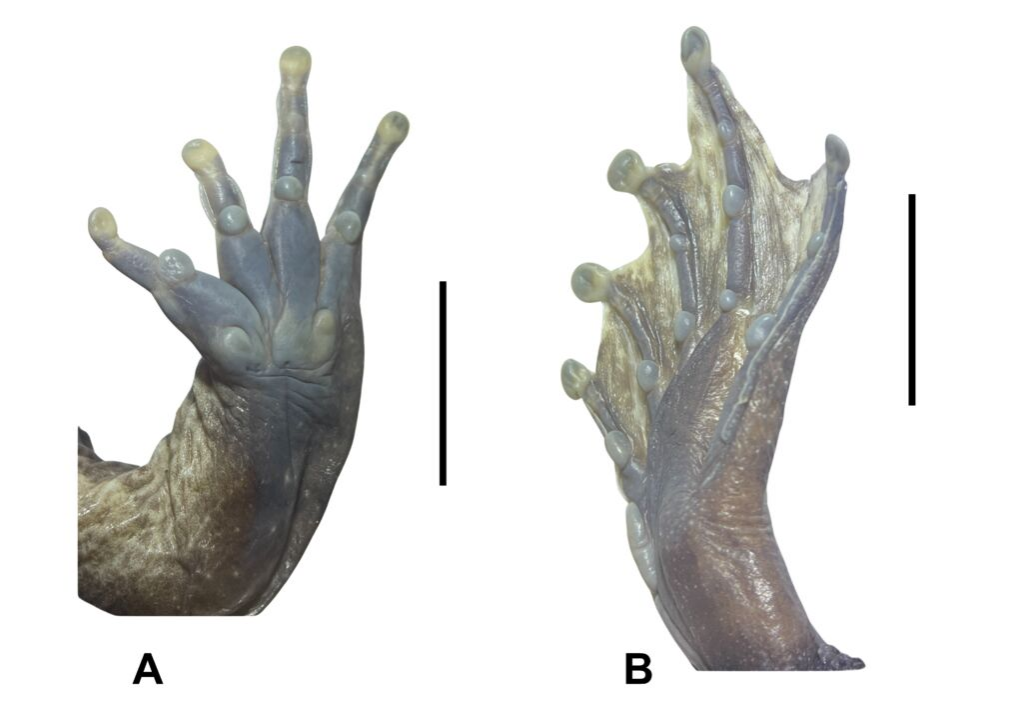
**A** lower right hand of *Limnonectesphuyenensis* (male, IEBR A.6348); **B** lower right foot of *Limnonectesphuyenensis* (male, IEBR A.6348).

Arms short, finger free of webbing, tips of fingers blunt, not expanded, fingers I with nuptial pad, composed of minute spines on dorsal surface and medial edge in males. Tibia shorter than thigh (FeL 29.2-31.7 mm, TbL 27.1-29.3 mm in males; 23.2 mm, 21.5 mm in female); tips of toes blunt, not expanded into discs toes webbed to distal of terminal phalanx; webbing well developed; tibio-tarsal articulation reaching to the tip of snout (Fig. 8).

Skin texture in life. Dorsal surface of head, body and flanks with ridges and tubercles; dorsal surface of tibia and foot distinctly tuberculate, covered with moderately dense, small, low tubercles; supratympanic fold present; dorsolateral fold absent.

Colouration in life. Dorsum yellowish-brown with dark brown markings; ventral surface white with brown markings (Fig. 7).

#### Distribution

In Vietnam, this species was previously known only from Phu Yen Province (Pham et al. 2020). This is the first record of *L.phuyenensis* from Khanh Hoa Province.

#### Ecology

The frogs were found between 19:00 and 22:30 h on the stones, on the ground or in the water of rocky streams. Surrounding habitat consisted of mixed secondary forest composed of large to medium hardwoods and shrubs. Air temperature was 22–29°C and relative humidity was 72–88%.

## Discussion

In terms of distribution, members of the genus *Limnonectes* are found in different geographical units in the Indochina Region (see Bain and Hurley (2011)): *Limnonectesbannaensis* is known from NWU, NEU, NEL and NAN; *L.dabanus* is known from CAN, CSL and SAN; *Limnonectesfastigatus* is known only from CAN; *L.gyldenstolpei* is known from NAN, UML and CMB; *L.kiziriani* is known only from CAN; *L.kohchangae* is known from CBM and MEK; *L.limborgi* is known from NWU, NEU, NEL, NAN, UML, SLU, SLL, CBM, CAN, CSL, SAN and MEK; *L.nguyenorum* is known from NWU, NEL and NEU; *L.phuyenensis* is known only from CAN; *L.poilani* is known from CAN, CBM, SAN and CSL; and *L.quangninhensis* is known from NEU and NIS (Table 2).

In terms of taxonomy, several populations of the *L.bannaensis* complex in Vietnam were described as new species, viz. *L.nguyenorum* from Ha Giang Province (McLeod et al. 2015), *L.quangninhensis* from Quang Ninh Province (Pham et al. 2017), *L.kiziriani* from Quang Binh, Thua Thien Hue and Gia Lai Provinces (Pham et al. 2018) and *L.phuyenensis* from Phu Yen Province (Pham et al. 2020).

The Annam Wart Frog (*Limnonectesdabanus*) was described from Lam Dong Province, Vietnam by Smith (1922) and is currently known from southern Vietnam and eastern Cambodia (Orlov et al. 2002, Nguyen et al. 2009). The Taylor's Frog (*Limnonecteslimborgi*) was described from Myanmar by Sclater (1892) and is currently known from Myanmar, Thailand, Malaysia, Laos, Vietnam and Cambodia (Frost 2025). The Phu Yen Wart Frog (*Limnonectesphuyenensis*) was described from Phu Yen Province by Pham et al. (2020), this species currently only known from Phu Yen Province, Vietnam, while the Virachey Fanged Frog (*Limnonectesfastigatus*) was described from Ratanakiri Province, north-eastern Cambodia by Stuart et al. (2020) and recorded from Vietnam for the first time by Pham et al. (2022).

In terms of conservation concern, one is listed as Vulnerable (VU) in the Red Data Book of Vietnam (Pham and Nguyen 2024) - *Limnonectesquangninhensis* is categorised as VU. Two species are listed in the IUCN Red List (IUCN 2024), including one species categorised as VU (*Limnonectesquangninhensis*) and one species categorised as NT (*Limnonectesnguyenorum*). Three species are currently known only from Vietnam, namely *Limnonecteskiziriani*, *L.phuyenensis* and *L.quangninhensis* (Table 2).

## Supplementary Material

XML Treatment for Limnonectes dabanus

XML Treatment for Limnonectes fastigatus

XML Treatment for Limnonectes limborgi

XML Treatment for Limnonectes phuyenensis

## Figures and Tables

**Table 1.** Measurements (in mm) of the specimens of *Limnonectes* from South Central Vietnam (abbreviations defined in the text).

	*Limnonectesdabanus*		*Limnonectesfastigatus*	*Limnonecteslimborgi*		*Limnonectesphuyenensis*	
	Male (n=2)	Females (n=2)	Male (n=2)	Male (n=1)	Female (n=3)	Male (n=4)	Female (n=1)
SVL	52.4-53.3	44.7-44.9	58.9-68.6	36.4	33-34.5	61.5-69.9	45.8
HL	23.5-27.3	18.2-18.3	26.3-29.8	15.5	12.5-14.2	28.5-32.2	19.9
HW	23.8-27.6	18.3-18.5	27.1-32.6	15.0	12.3-13.7	29.7-33.3	20.5
RL	8.5-9.5	6.8-7.1	8.5-10.2	6.0	5.0-5.3	8.6-9.9	7.0
ED	5.7-5.9	5.1-5.2	7.1-8.0	4.0	4.3-4.5	7.2-8.4	5.9
UEW	2.9-3.5	3.1-3.2	3.6-3.8	2.6	2.5-3.3	3.5-4.3	2.8
IND	5.3-5.9	4.3-4.5	6.0-6.2	4.6	4.0-4.5	5.5-6.3	4.9
IOD	4.6-4.8	3.5-3.7	5.6-5.8	3.6	3.0-3.2	5.4-5.8	3.7
NS	3.4-4.4	2.8-3.1	4.1-4.8	3.1	2.3-2.6	3.4-4.8	3.0
EN	4.9-5.3	3.8-3.9	4.4-5.4	2.9	2.8-2.9	4.4-5.0	3.9
TD	5.2-5.3	3.4-3.5	NA	2.1	2.7-2.8	NA	NA
TYE	4.0-4.4	2.6-2.8	NA	2.2	1.5-1.8	NA	NA
UAL	10.6-12.3	9.1-9.6	11.8-14.3	7.3	7.1-7.4	11.0-12.6	8.6
FAL	22.1-22.2	19.8-20	23.1-29.1	14.6	12.0-13.6	25.1-29.2	20.5
FeL	23.9-24.2	21.1-21.7	27.2-31.1	18.8	16.5-17.4	29.2-31.7	23.2
TbL	26.3-26.5	22.0-22.8	26.1-29.8	19.2	17.6-18.0	27.1-29.3	21.5
TbW	8.1-8.3	6.5-6.9	10.8-12.8	5.4	4.2-4.8	13.0-14.2	9.2
FoL	25.4-25.5	21.6-22.9	32.5-38.6	27.7	24.5-25.1	36.0-37.5	28.7
HL/SVL	0.45-0.51	0.41-0.41	0.43-0.45	0.43	0.37-0.41	0.45-0.48	0.43
HW/SVL	0.44-0.48	0.4-0.41	0.46-0.48	0.41	0.36-0.40	0.47-0.51	0.45
RL/SVL	0.16-0.18	0.15-0.16	0.14-0.15	0.16	0.15-0.16	0.14-0.15	0.15
HL/HW	1.02-1.06	1.01-1.03	0.91-0.97	1.03	1.02-1.06	0.94-0.97	0.97
ED/RL	0.60-0.68	0.73-0.75	0.78-0.84	0.67	0.83-0.9	0.75-0.93	0.84
TYE/TD	0.70-0.74	0.74-0.82	NA	1.05	0.56-0.67	NA	NA
TD/ED	0.90-0.91	0.85-0.88	NA	0.53	0.6-0.63	NA	NA
TbL/SVL	0.49-0.51	0.49-0.51	0.43-0.44	0.53	0.52-0.53	0.43-0.48	0.49
TbL/TbW	3.17-3.27	3.30-3.38	2.33-2.42	3.56	3.67-4.26	2.10-2.26	2.46

**Table 2.** List of genus Limnonectes recorded from Vietnam. RBVN (2024) = Vietnam Red Data Book VU = Vulnerable (Pham and Nguyen 2024); IUCN (2024) = The IUCN Red List of Threatened Species. VU = Vulnerable, NT = Near Threatened. Bain and Hurley (2011): NWU = Northwest Uplands; NEU = Northeast Uplands; NAN = Northern Annamites; NEL = Northeast Lowlands; UML = Upper Mekong Lowlands; CAN = Central Annamites; SAN = Southern Annamites; CSL = Central-South Vietnam Lowlands; SLU = Southern Lao Uplands; SLL = Southern Lao Lowlands; MEK = Mekong Delta; CMB = Interior Cambodian Lowlands; and NIS = Northern Islands.

Name	RBVN 2024	IUCN (2024)	Distribution by subregions of Indochina (Bain and Hurley 2011)
*Limnonectesbannaensis* Ye, Fei, Xie & Jiang, 2007			NWU, NEU, NEL, NAN
*Limnonectesdabanus* (Smith, 1922)			CAN, CSL, SAN
*Limnonectesfastigatus Stuart*, Schoen, Nelson, Maher, Neang, Rowley & McLeod, 2020			CAN
*Limnonectesgyldenstolpei* (Andersson, 1916)			NAN, UML, CMB
*Limnonecteskiziriani* Pham, Le, Ngo, Ziegler & Nguyen, 2018			CAN
*Limnonecteskohchangae* (Smith, 1922)			CBM, MEK
*Limnonecteslimborgi* (Sclater, 1892)			NWU, NEU, NEL, NAN, UML, SLU, SLL, CBM, CAN, CSL, SAN, MEK
*Limnonectesnguyenorum* McLeod, Kurlbaum & Hoang, 2015		NT	NWU, NEL, NEU
*Limnonectesphuyenensis* Pham, Do, Le, Ngo, Nguyen, Ziegler & Nguyen, 2020			CAN
*Limnonectespoilani* (Bourret, 1942)			CAN, CBM, SAN, CSL
*Limnonectesquangninhensis* Pham, Le, Nguyen, Ziegler, Wu & Nguyen, 2017	VU	VU	NEU, NIS
